# Combatting Substandard and Falsified Medicines: Public Awareness and Identification of Counterfeit Medications

**DOI:** 10.3389/fpubh.2021.754279

**Published:** 2021-10-26

**Authors:** Faris El-Dahiyat, Khairi M. S. Fahelelbom, Ammar Abdulrahman Jairoun, Sabaa Saleh Al-Hemyari

**Affiliations:** ^1^College of Pharmacy, Al-Ain University, Al Ain, United Arab Emirates; ^2^Health and Safety Department, Dubai Municipality, Dubai, United Arab Emirates; ^3^Pharmacy Department, Emirates Health Services Establishment, Dubai, United Arab Emirates

**Keywords:** falsified mediation, counterfeit medications, knowledge–attitude–behavior, identification, public awareness

## Abstract

**Objectives:** The objective of this study was to determine the identification rate of substandard and falsified medications and its association with knowledge among public.

**Methods:** This descriptive cross-sectional study was conducted in different geographic areas among a convenient sample of people aged 18 or older. A validated web-based electronic questionnaire was used for data collection tool. The questionnaire contained three sections assessing the following: (1) Sociodemographic data; (2) Knowledge regarding counterfeit medicines; and (3) Ability to identify counterfeit medicines, according to 12 questions rated on a five-point Likert scale. Univariate and multivariate logistic regression analyses were used to assess the association between sociodemographic factors and counterfeit medication identification rate.

**Results:** A total of 320 people participated in the study. Only 98 participants (30.6%, 95% CI 25.6–35.7%) identified the counterfeit medications. Ability to correctly identify counterfeit medications was significantly higher in participants who were older (*p* = 0.016), single (*p* = 0.001), Asian (*p* = 0.001), or American (*p* = 0.019), as well as those who indicated that they would check the certification of the medications (*p* = 0.015) and report counterfeit medications to the authorities (*p* < 0.0001).

**Conclusions:** These results underscore the need for greater public awareness of the hazards associated with counterfeit medicines.

## Introduction

The World Health Organization (WHO) defined counterfeit pharmaceutical products as those that are deliberately and fraudulently mislabelled with respect to identity and/or source (1). Recently, WHO has introduced the more specific term “substandard and falsified (SF) medical products.” Substandard medical products—also called “out of specification” products—are authorized medical products that fail to meet necessary quality standards or specifications. Unregistered or unlicensed medical products that have not undergone evaluation or approval by the National or Regional Regulatory Authority (NRRA) for the market in which they are marketed or distributed are subject to permitted conditions under national or regional regulations and legislation. On the other hand, falsified medical products are ones that deliberately or fraudulently misrepresent their identity and composition or source (2, 3).

WHO has determined that counterfeit products could comprise about 50% of the drug market worldwide; many of these products arise from developing countries (4). According to reports sent to WHO from 20 different countries, most falsified drugs fell into one the following three categories: (1) products containing no active ingredient: about 30%; (2) products containing an incorrect quantity of the active ingredient: about 20%; and (3) products containing wrong contents: about 20% (5). According to an estimate, one in 10 pharmaceutical products are substandard or even falsified in low- and middle-income countries. Antimalarial medications are the most taking place counterfeit drugs, exhibiting about 20 per cent of the total counterfeit products and drugs reported in the year 2017 (6). The predominance of counterfeit products is highest in developing countries in Africa, Asia, and Latin America, comprising about 30–60% of all drugs in the market (2, 7–14). Worldwide, it is estimated that 10–15% of drugs are counterfeit (2, 7). Approximately 35–75% of the fake or counterfeit products that arise globally are made in India (11, 12).

Stricter methodological standards are required to determine the scale of this problem in order to raise awareness and ultimately address it. Global collaborative actions are required to enhance the management and surveillance of pharmaceutical supply-chains and regulatory agencies in low- and middle-income countries to minimize the threat of poor-quality drugs (15).

Counterfeit products are hazardous to health as well as the economy, and this is a dilemma for almost all developed and developing countries around the world. Besides. they are a major cause of treatment failure, adverse events, mortality, economic strain, development of drug resistance, and loss of confidence in medicine and various health services (3, 9, 13). The economic burden of counterfeit medicines is considerable in many developing countries, as illustrated by lost sales revenues, lost tax profit, job loss, increasing spending to fight and root out counterfeit products, money spent on ineffective counterfeit products, and expenses associated with recurrent hospitalizations due to counterfeit drugs (16–18).

Factors that increase a country's susceptibility to counterfeit medicines include healthcare infrastructure collapse, inadequate or improper regulatory procedures, and excessive costliness of essential medicines (18). According to a study in Nigeria, factors that contribute to the use of counterfeit medicines include illiteracy, ignorance, and weak law enforcement (19). According to this study, the main reasons for the prevalence of counterfeit medicine were the high cost of drugs and the greed of regulatory officials (19). From a public health perspective, counterfeit drugs threaten to deplete resources and result in poor patient outcomes, drug toxicity, disability, and even mortality. Moreover, from a commercial perspective, counterfeit drugs raise the issue of patent law violations (20).

WHO and other organizations have argued for increased public awareness of the flourishing counterfeit drugs trade and its associated public health risks (21, 22). There is limited data regarding people's awareness of counterfeit drugs and their ability to identify them.

A counterfeit drug might look similar to the original product, and, unfortunately, the only way to conclusively verify whether a product is fake is through chemical testing in an officially licensed laboratory. That said, physical evaluation of a drug's label and package design can provide clues into its counterfeit status. At a minimum, the label of any drug product (including even dietary supplements) is mandated to include: the brand name of the product, the drug's generic name, indications, size/weight, warnings/cautions, usage instructions, manufacturing details, country of origin, batch number, and a clear barcode. The absence of any of these items is a crucial indicator of counterfeit products. Awareness programs about these drug labeling requirements would be a vital step in combatting counterfeit products and limiting their use. The prevalence of low-quality drugs is a vital but poorly studied issue.

Hence this study aims to determine the identification rate of substandard and falsified medications and its association with knowledge among public.

## Materials and Methods

### Study Population

This cross-sectional study was conducted using online survey in different geographic areas Europe, Asia, Africa, America and Middle East through a convenience sample of participants with different educational levels (e.g., high school, Bachelor's degree and Post graduate who were willing to participate in the study). Data was collected between May and November 2019.

### Ethical Consideration

The study was approved by the Institutional Ethical Review Committee of Al Ain University (COP/2019/33) and conducted in accordance with the Declaration of Helsinki (23). The nature and purpose of the study was explained on first page of the online survey, where the participation in the survey and the individual responses will be strictly confidential to the research team and will not be divulged to any outside party. The informed consent was obtained by a participant's choice to continue to the next page and was considered their consent to participate in the study.

### Data Collection

An online link to our survey was sent by email to valid and active professional LinkedIn accounts and universities websites within and outside the UAE. Accounts were retrieved from the web browser Google and recorded in a spreadsheet. Accounts were subsequently classified as personal or belonging to university institutions, private institutions, or government institutions. This online data collection method considered convenient and was used to reduce survey bias from “interviewer effect,” to ease of allowing only the targeted demographics to participate, and ease of screening participants.

### Sample Size Calculation

Since the estimated awareness of counterfeit medicines ranges from 50 to 93% (24–27), we assume 50% prevalence of public awareness about counterfeit medicines. Using an alpha level of 5% and a 95% confidence interval, we determined that a sample size of 550 participants is necessary for this study, assuming a non-response rate of 30%. The response rate was calculated from the pilot study. The survey link questionnaire was sent to 30 participants, from which 21 returned the filled questionnaire (response rate 70%). The following sample equation was used:


$$\begin{array}{l} n=\frac{\left(\text{Z}\right)\;2{\;}^{*}\left(\text{P}\right){\;}^{*}\;\left(1-\text{P}\right)}{\left(\text{E}\right)\;2} \end{array}$$

### Research Instrument Development

The study questionnaire was developed based on items used in previous questionnaires (24–27). The questionnaire was subsequently validated and examined for the relevance and appropriateness of its contents by two clinical pharmacy lecturers from Al Ain University. In addition, Lawshe's method of assessing content validity was used to assess the instrument's quantitative content validity. Only the items with a content validity ratio (CVR) score ≥ 0.78 were included in the instrument. The instrument's calculated Cronbach's α value (0.72) demonstrated that it has an acceptable degree of internal consistency. The validated version of the questionnaire was then piloted with 30 subjects to ensure relevance and clarity. Responses from the pilot stage were not included in the final study results.

### Research Instrument Sections

The questionnaire contained three sections assessing the following: (1) Sociodemographic data (e.g., sex, age, marital status, geographic area, education level, employment status, chronic disease diagnoses, and medication history); (2) Knowledge regarding counterfeit medicines; and (3) Ability to identify counterfeit medicines. The third section was comprised of 12 questions in which participants were asked to indicate how often they check the different pieces of information on a drug's label when purchasing medication. This was rated on a five-point Likert scale (1 = “never,” 2 = “rarely,” 3 = “sometimes,” 4 = “often,” 5 = “always”).

### Statistical Analysis

The analysis was performed using SPSS version 24. Qualitative variables were summarized using frequencies (percentages) as appropriate, while quantitative variables were summarized using means and standard deviations (SDs). 12 Questions addressing the counterfeit mediation identification could be answered by “always,” “often,” “sometimes,” “rarely,” or “never.” A counterfeit mediation identification score was created to measure the awareness and ability of participants in identifying counterfeit medication. This score was dichotomized into two categories (e.g., yes and no). Correct response (yes) scored 1 and wrong answer (no) scored “0.” The participant considered has awareness about counterfeit identification if he correctly identified all the 12 items.

Chi-square and fisher exact tests were used to determine the difference in the identification rate of substandard and falsified medications according to demographics. Univariate and multivariate logistic regression analyses were used to assess the association between sociodemographic factors and counterfeit medication identification rate. *P*-values <0.05 were considered statistically significant.

## Results

### Demographic Characteristics of the Study Population

A total of 320 subjects participated in the study and completed the whole questionnaire. Among these 55% (*n* = 176) were male and 45% (*n* = 144) were female. Of the total participants, 11.3% (*n* = 36) were ages 18–24, 21.9% (*n* = 70) were ages 25–34, 20.6% (*n* = 66) were ages 35–44, 31.9% (*n* = 102) were ages 45–54, and 14.4% (*n* = 46) were ages ≥55. A majority of the participants were married (*n* = 250, 78.1%). A majority of the participants were from the Middle East (58.8%), followed by Africa (23.8%), Europe (7.5%), Asia (6.9%), and America (3.1%). About 66.3% of the subjects were high school educated, whereas 31.3% were bachelor's degree holders and 2.5% were graduate degree holders. A majority of the participants were employed (75%). Overall, 70 (21.9%) participants reported having a chronic disease and 266 (83.1%) were taking medication for acute disorders (Table 1).

**Table 1:** Frequency table for demographic characteristics (*n* = 320).

**Variable**	**Groups**	**Frequency**	**Percentage**
Sex	Male	176	55%
	Female	144	45%
Age	18–24	36	11.3%
	25–34	70	21.9%
	35–44	66	20.6%
	45–54	102	31.9%
	≥55	46	14.4%
Marital status	Single	70	21.9%
	Married	250	78.1%
Regions	Europe	24	7.5%
	Asia	22	6.9%
	Africa	76	23.8%
	America	10	3.1%
	Middle East	188	58.8%
Education	Post graduate	8	2.5%
	Bachelor‘s degree	100	31.3%
	High school	212	66.3%
Employment status	Employed	240	75%
	Unemployed	80	25%
Chronic disease	Yes	70	21.9%
	No	250	78.1%
Take Medication in case of acute disorders	Yes	266	83.1%
	No	54	16.9%

### Knowledge and Current Practice Toward Counterfeit Medications

Study participants' knowledge and current practice toward counterfeit medications are summarized in Table 2. Out of all participants, 51.9% were aware of the hazards of counterfeit medications and 83.8% disagreed with the statement that counterfeit medications are harmless. In this study, 120 (37.5%) of the respondents believed that counterfeit medicines constitute a national health problem in their countries and 188 (58.8%) indicated that it is important to ensure that medicines were certified and registered by a regulatory authority. As for participants' personal experiences with counterfeit medications, 286 (89.4%) never or rarely encountered them, 26 (8.1%) sometimes encountered them, and 8 (2.5%) often or always encountered them. Only eight participants (2.5%) had ever reported counterfeit medications to the relevant authority.

**Table 2:** Frequency table for knowledge and practice items.

**Knowledge and practice items**	**Response**	**Frequency**	**Percentages**
Are you aware of any Hazards that might be associated with the use of the Counterfeit medications	Yes	166	51.9%
	No	154	48.1%
Do you agree with the statement that Counterfeit medications are harmless	Disagree	268	83.8%
	Neutral	16	5%
	Agree	36	11.3%
Counterfeit medicines are a national health problem in your country	Disagree	68	21.3%
	Neutral	132	41.3%
	Agree	120	37.5%
Do you make sure that the medication is certified by the regulated authority	Yes	188	58.8%
	No	132	41.3%
How frequently have you encountered Counterfeit medications?	Never/Rarely	286	89.4%
	Sometimes	26	8.1%
	Always/Often	8	2.5%
Have you ever reported the Counterfeit medications to concerned authority	Yes	8	2.5%
	No	312	97.5%

### Identification of Counterfeit Medications

In this study, 98 participants (30.6%, 95% CI 25.6–35.7%) correctly identified the counterfeit medications. Table 3 shows participant's responses to the counterfeit medications' identification items. The most often identified piece of label information was production date or expiration date (4.53 ± 0.82), followed by instructions of use (4.38 ± 0.94), and the generic drug name (4.1 ± 1.17). The least often identified pieces of label information were batch number (1.58 ± 0.97), barcode (1.73 ± 1.1), and manufacturing details (2.55 ± 1.31) (Table 3).

**Table 3:** Frequency table for counterfeit identification items (*n* = 320).

**Counterfeit identification item**	**Never *n* (%)**	**Rarely *n* (%)**	**Sometimes *n* (%)**	**Often *n* (%)**	**Always *n* (%)**	**Mean ± (SD)**
Generic name	18 (5.6%)	20 (6.3%)	42 (13.1%)	88 (27.5%)	152 (47.5%)	4.05 (±1.17)
Trade name	26 (8.1%)	32 (10%)	64 (20%)	62 (19.4%)	136 (42.5%)	3.78 (±1.31)
Net weight/Size/dose	28 (8.8%)	38 (11.9%)	48 (15%)	60 (18.8%)	146 (45.6%)	3.81 (±1.36)
Indication	6 (1.9%)	16 (5%)	46 (14.4%)	76 (23.8%)	176 (55%)	4.25 (±1)
Usage/how to use	4 (1.3%)	18 (5.6%)	26 (8.1%)	76 (23.8%)	196 (61.3%)	4.38 (±0.94)
Cautions/warnings	10 (3.1%)	30 (9.4%)	50 (15.6%)	112 (35%)	118 (36.9%)	3.93 (±1.1)
Storage conditions	16 (5%)	48 (15%)	76 (23.8%)	88 (27.5%)	92 (28.7%)	3.6 (±1.2)
Country of origin	22 (6.9%)	54 (16.9%)	70 (21.9%)	92 (28.7%)	82 (25.6%)	3.49 (±1.2)
Manufacturing details	92 (28.7%)	72 (22.5%)	80 (25%)	44 (13.8%)	32 (10%)	2.55 (±1.31)
Barcode	184 (57.5%)	80 (25%)	26 (8.1%)	16 (5%)	14 (4.4%)	1.73 (±1.1)
Batch number	208 (65%)	66 (20.6%)	28 (8.8%)	8 (2.5%)	10 (3.1%)	1.58 (±0.97)
Production/Expiry date	6 (1.9%)	4 (1.3%)	20 (6.3%)	74 (23.1%)	216 (67.5%)	4.53 (±0.82)

*n, frequency; %, percentage*.

Table 4 presents the counterfeit medications identification rate according to demographic factors. Participants who were male (*p* = 0.048), older (*p* = 0.006), single (*p* = 0.012), and highly educated had a significantly higher counterfeit medication identification rate. Moreover, there was a significant association between geographic regions and counterfeit medication identification rate (*p* = 0.021). Participants from the Middle East had a significantly lower counterfeit medication identification rate compared to those from other regions.

**Table 4:** Counterfeit identification according to demographic factors.

	**Proportional of awareness about counterfeit identification**		
**Variable**	**Groups**	**Frequency (%)**	***P*-value**
Sex	Male	62 (35.2%)	0.048
	Female	36 (25%)	
Age	18–24	14 (38.9%)	0.006
	25–34	28 (40%)	
	35–44	14 (21.2%)	
	45–54	22 (21.6%)	
	≥55	20 (43.5%)	
Marital status	Single	30 (42.9%)	0.012
	Married	68 (27.2%)	
Regions	Europe	8 (33.3%)	0.021
	Asia	12 (54.5%)	
	Africa	22 (28.9%)	
	America	6 (60%)	
	Middle East	50 (26.6%)	
Education	High school	62 (29.2%)	0.022
	Bachelor‘s degree	30 (30%)	
	Postgraduate	6 (75%)	
Employment status	Employed	74 (30.8%)	0.889
	Unemployed	24 (30.0%)	
Chronic disease	Yes	20 (28.6%)	0.673
	No	78 (31.2%)	
Take Medication in case of acute disorders	Yes	80 (30.1%)	0.636
	No	18 (33.3%)	

*p-values reported above are for comparisons between variable levels “categories–levels” using the chi-square and fisher exact tests*.

Table 5 shows the association between the counterfeit medication identification rate and participants' knowledge and practice toward counterfeit medications. Participants who reported checking the certification medications (*p* = 0.002), those who frequently encountered counterfeit medications (*p* = 0.003), and those who reported the counterfeit medications to the relevant authorities (*p* < 0.0001) had a significantly higher counterfeit medication identification rate.

**Table 5:** Counterfeit identification according to knowledge and practice factors.

**Knowledge and practice items**	**Proportional of awareness about counterfeit identification**		
	**Response**	**Frequency (%)**	***P*-value**
Are you aware of any hazards that might be associated with the use of the Counterfeit medications	Yes	52 (31.3%)	0.778
	No	46 (29.9%)	
Do you agree with the statement that Counterfeit medications are harmless	Disagree	80 (29.9%)	0.479
	Neutral	4 (25%)	
	Agree	14 (38.9%)	
Counterfeit medicines is a national health problem in your country	Disagree	26 (38.2%)	0.253
	Neutral	40 (30.3%)	
	Agree	32 (26.7%)	
Do you make sure that the medication is certified by the regulated authority	Yes	70 (37.2%)	0.002
	No	28 (21.2%)	
How frequently have you encountered Counterfeit medications	Never/Rarely	80 (28.0%)	0.003
	Sometimes	12 (46.2%)	
	Always/Often	6 (75%)	
Have you ever reported the Counterfeit medications to concerned authority	Yes	8 (100%)	<0.0001
	No	90 (28.8%)	

*p-values reported above are for comparisons between variable levels “categories–levels” using the chi-square and fisher exact tests*.

### Factors Associated With Counterfeit Mediation Identification Rate

We performed a univariate and multivariate logistic regression analysis to identify factors that were highly correlated with counterfeit medication identification rate. The univariate analysis showed that the following factors significantly increased the counterfeit medication identification rate: male sex (OR 3.61, 95% CI 2.52–5.16), older age (OR 1.93, 95% CI 1.02–3.66), single marital status (OR 2.01, 95% CI 1.16–3.48), Asian geography (OR 3.31, 95% CI 1.35–8.14), American geography (OR 4.14, 95% CI 1.12–7.28), graduate education (OR 7.26, 95% CI 1.43–11.95), a habit of checking the certification of medications (OR 2.20, 95% CI 1.32–3.67), frequent encountering of counterfeit medications (OR 7.73, 95% CI 1.53–12.1), and reporting counterfeit medications to the relevant authorities (OR 6.32, 95% CI 1.43–14.23).

In multivariate analysis, counterfeit medication identification rate was significantly associated with older age (*p* = 0.016), being single (*p* = 0.001), Asian geography (*p* = 0.001), American geography (*p* = 0.019), a habit of checking the certification of medications (*p* = 0.015), and reporting counterfeit medications to the relevant authorities (*p* < 0.0001) (Table 6).

**Table 6:** Univariate and multivariate analysis of factors associated with counterfeit identification.

**Factors**	**Counterfeit identification**							
	**Univariate**				**Multivariate**			
	**OR**	**95% CI**		***P*-value**	**OR**	**95% CI**		***P*-value**
Female	Ref.				—	—	—	—
Male	1.63	1.002	2.66	0.049	—	—	—	—
<55 years	Ref.				Ref.			
≥55 years	1.93	1.02	3.66	0.043	2.46	1.184	5.104	0.016
Married	Ref.				Ref.			
Single	2.01	1.16	3.48	0.013	3.200	1.651	6.201	0.001
Middle East	Ref.							
Europe	1.38	0.556	3.42	0.487	1.658	0.620	4.435	0.314
Asia	3.31	1.35	8.14	0.009	5.150	1.930	10.75	0.001
Africa	1.124	0.622	2.03	0.698	1.559	0.784	3.099	0.205
America	4.14	1.12	7.28	0.03	5.710	1.338	8.372	0.019
High school	Ref.				—	—	—	—
Bachelor‘s degree	1.04	0.62	1.74	0.891	—	—	—	—
Postgraduate	7.26	1.43	11.95	0.017	—	—	—	—
Do you make sure that the medication is certified by the regulated authority								
No	Ref.							
Yes	2.20	1.32	3.67	0.002	1.99	1.144	3.48	0.015
Encountered Counterfeit medications								
Never/Rarely	Ref.				—	—	—	—
Sometimes	2.21	0.98	4.978	0.056	—	—	—	—
Always/Often	7.73	1.53	12.074	0.013	—	—	—	—
Reported the Counterfeit medications to concerned authority								
No	Ref.				Ref.			
Yes	6.32	1.43	14.23	0.000	4.56	1.78	12.98	0.000

*P-values <0.05 were considered statistically significant, “—” not included in the multivariate logistic regression model. OR, odds ratio; CI, confidence interval*.

## Discussion

This study explored public knowledge and practice toward counterfeit drugs using a cross-sectional study design. Over half of the participants (51.9%) were aware of the hazards of counterfeit medications, and the vast majority (83.8%) disagreed with the statement that counterfeit medications are harmless. In contrast, a previous study of Italian healthcare professionals reported much lower knowledge of the characteristics of falsified medicines: Between 25 and 50% of the respondents were unaware that counterfeit medications could contain additives, a lower dose of the active ingredient, and/or incorrect ingredients, and only a quarter of the respondents knew that counterfeit drugs could be lethal, this difference between our study and the Italian study could possibly be due to the fact that Legal online sale of medicines in Italy has started being regulated only recently. In addition, awareness of our respondents is clearly higher (28).

Few studies in the literature have addressed public awareness and knowledge of counterfeit medicine. The few that have focused mostly on exploring and combating counterfeit drugs in the developing world. 93.4% of respondents in a Lebanese study showed they were aware of the term counterfeit medicines. Another Lebanese qualitative study demonstrated a gap in participants' awareness regarding counterfeit medicines, and the majority of participants could not define counterfeit medicines. In Qatar, a similar study revealed that pharmacists had higher awareness than public regarding counterfeit medicines and its societal consequences.

In general, our results are in line with other studies from different countries regrading public knowledge and practice toward counterfeit medications (25–30).

Whereas in our study 30.6% (95% CI 25.6–35.7%) of the participants could correctly identify counterfeit medications, a similar study conducted in England (*n* = 320) reported that 62.8% of the participants were aware of the presence of falsified products purchased online and 11.9% had encountered counterfeit items, of whom only 0.9% reported the counterfeit items to the authorities (31, 32).

We found that more highly educated participants had a higher counterfeit medication identification rate. Furthermore, participants from countries in the Middle East had a significantly lower counterfeit medication identification rate compared to participants from other regions. A previous study on public awareness and identification of counterfeit drugs in Tanzania reported that 55.6% of participant were able to distinguish between a genuine vs. counterfeit drugs while 44.4% failed to identify (24, 33).

Counterfeit medicine identification rate in our study was significantly associated with older age (*p* = 0.016), single marital status (*p* = 0.001), Asian geography (*p* = 0.001), and American geography (*p* = 0.019). In contrast, in the Tanzanian study, age, sex, education level, and marital status were not associated with ability to identify counterfeit drugs (24).

Overall, this study highlights the need for awareness and educational campaigns about the safety and efficacy of medicines, and the importance of avoiding counterfeit drugs. Healthcare providers must be actively involved in educating patients and the public on the adverse consequences of counterfeit drugs and measures to identify them. Pharmacists, doctors, and nurses—in addition to pharmaceutical companies and medication distributors—should be constantly vigilant about drug falsification so that they are prepared to deal with suspicious drug products (22).

## Conclusion

Drug counterfeiting is a threat to every nation's public health and economy. Only 30.6% of the participants able to identify properly the counterfeit medications. There is a dire need to raise awareness among general population through different awareness and educational campaigns to help identifying counterfeit drug products. The involvement of healthcare professionals is crucial in this regard.

## Data Availability Statement

The raw data supporting the conclusions of this article will be made available by the authors, without undue reservation.

## Ethics Statement

The studies involving human participants were reviewed and approved by Al Ain University (COP/2019/33). The patients/participants provided their written informed consent to participate in this study.

## Author Contributions

FE-D and KF designed the study. FE-D, AJ, and SA-H responsible for data collection. FE-D and AJ analyzed and interpreted the data. All authors drafted, reviewed, and approved the manuscript.

## Conflict of Interest

The authors declare that the research was conducted in the absence of any commercial or financial relationships that could be construed as a potential conflict of interest.

## Publisher's Note

All claims expressed in this article are solely those of the authors and do not necessarily represent those of their affiliated organizations, or those of the publisher, the editors and the reviewers. Any product that may be evaluated in this article, or claim that may be made by its manufacturer, is not guaranteed or endorsed by the publisher.
